# Quercetin may reduce the risk of developing the symptoms of COVID-19

**DOI:** 10.22038/AJP.2023.22920

**Published:** 2024

**Authors:** Marjan Ajami, Mohammadjavad Sotoudeheian, Anahita Houshiar-Rad, Mina Esmaili, Fatemeh Naeini, Fatemeh Mohammadi Nasrabadi, Saied Doaei, Ali Milani-Bonab

**Affiliations:** 1 *Department of Food and Nutrition Policy and Planning Research, Faculty of Nutrition Sciences and Food Technology, National Nutrition and Food Technology Research Institute, Shahid Beheshti University of Medical Sciences, Tehran, Iran*; 2 *Physiology Research Center, Faculty of Medicine, Iran University of Medical Sciences, Tehran, Iran*; 3 *Department of Nutrition Research, Faculty of Nutrition Sciences and Food Technology, National Nutrition and Food Technology Research Institute, * *‎* *Shahid Beheshti University of Medical Sciences, Tehran, Iran* *‎*; 4 *Department of Clinical Nutrition, School of Nutritional Sciences and Dietetics, Tehran university of Medical Science, Tehran, Iran*; 5 *Department of Community Nutrition, Faculty of Nutrition and Food Technology, National Nutrition and Food Technology Research Institute, Shahid Beheshti University of Medical Sciences, Tehran, Iran*

**Keywords:** COVID-19, SARS-CoV-2 main protease, ACE-2 receptors, RNA-dependent RNA polymerase, Quercetin

## Abstract

**Objective:**

Recent evidence reported that some dietary compounds like quercetin and apigenin as the most well-known flavonoids with anti-inflammatory effects may inhibit SARS-CoV-2 main protease. The hypothesis of the promising effects and possible mechanisms of action of quercetin against COVID-19 were assessed in this article.

**Materials and Methods:**

Related papers on the inhibitory effects of quercetin against COVID-19 were collected using the following search strategy: “corona or coronavirus or COVID or COVID-19 or viral or virus” AND “nutrient or flavonoid or Quercetin”.

**Results:**

The findings indicated that quercetin can be considered an effective agent against COVID-19 because of its SARS-CoV-2 main protease and RNA-dependent RNA polymerase inhibitory effects. In addition, quercetin may attenuate angiotensin-converting enzyme-2 (ACE-2) receptors leading to a reduction of SARS-CoV-2 ability to enter host cells. Moreover, the antiviral, anti-inflammatory, and immunomodulatory activities of quercetin have been frequently reported.

**Conclusion:**

Quercetin may be an effective agent for managing the complications of COVID-19. Further longitudinal human studies are warranted.

## Introduction

From 2019, a novel severe acute respiratory syndrome coronavirus 2 (SARS-CoV-2) brought about the COVID-19 pandemic representing an emergent concern worldwide (Lai et al., 2020) . The World Health Organization (WHO) reported more than six million deaths worldwide caused by COVID-19 until April, 2022(Organization, 2022) . SARS-CoV-2 is now considered a new human pathogen contributing to severe acute respiratory syndrome (SARS) (Zheng, 2020) . In the last two decades, several viral diseases had been reported, e.g. SARS in Guangdong province, China (2002), and Middle East respiratory syndrome coronavirus (MERS-CoV) in Saudi Arabia (2012) (da Costa et al., 2020) . However, this new virus belonging to the coronavirus (CoV) family has a much faster communicable capacity compared to the SARS and MERS coronaviruses (Chen, 2020) .

Currently, there are no known antiviral drugs available against the COVID-19 (Abd El-Aziz and Stockand, 2020; Edwin and Antony, 2023; Yip et al., 2023) . Recent publications suggested a combination of antiviral drugs such as remdesivir (Wang et al., 2020) , Pfizer’s Paxlovid and Merck’s Molnupiravir{Wen, 2022 #72;Abd El-Aziz, 2020 #2415} , along with preventive policies like social distancing, isolating ill people, washing hands, and self-quarantine as potent strategies against COVID-19 (Wilder-Smith and Freedman, 2020) . Also, focusing on appropriate symptomatic management in patients with SARS-CoV-2 is highly recommended (Zhang et al., 2020) . Some of the most common symptoms are fever, cough, pneumonia, myalgia, and complex dyspnea (Jiang et al., 2020) . Older adults and people with underlying chronic diseases such as diabetes, hypertension, cardiovascular disease, malignancies, and chronic inflammation which affect immunological responses, including the release of various cytokines are at a much higher risk of COVID-19 (Hussain et al., 2020) . Angiotensin-converting enzyme-2 (ACE-2), an emerging target for treatment of acute respiratory distress syndromes (ARDS), was identified as a specific receptor for SARS-CoV-2 (Gheblawi et al., 2020) . The virus infects the cells through ACE-2 and stimulates cytokine storms leading to serious damages to the tissues (Song et al., 2020) . SARS-CoV-2-infected cells produce excessive levels of angiotensin II leading to the down-regulation of ACE-2 expression (Verdecchia et al., 2020) . It has postulated that the reduction of ACE-2 activity in host-cell membranes may restrict the ability of SARS-CoV-2 to enter cells (Zemlin and Wiese, 2020) . 

Some studies indicated the possible anti-inflammatory effects of quercetin which may be served as an effective option against some complications of COVID-19 (Pooladanda et al., 2020) . Also, recent evidence reported that some flavonoids including quercetin, naringenin, kaempferol, and apigenin may potentially inhibit SARS-CoV-2 main protease (Khaerunnisa et al., 2020) . The COVID-19 main protease plays crucial role in CoV replication; thus, preventing the enzymatic activity of the COVID-19 protease may lead to favorable effects against COVID-19 (Tutunchi et al., 2020) . Production of effective and appropriate drugs focusing on ACE-2 and SARS-CoV-2 main protease can be an attractive and efficient field in the management of CoV(Liu et al., 2020b). Accordingly, this study aimed to assess the literature on the promising effects and possible mechanisms of action of quercetin, as a bioactive flavonol against coronavirus disease. The present manuscript hypothesizes that quercetin as a polyphenolic flavonol might offer favorable effects against COVID-19

## Materials and Methods

Relevant peer-reviewed articles published in English up to August 2022 were selected for this study by searching reputable databases including PubMed, Scopus, and Web of Science. The current research was performed using the terms of medical subject headings (MeSH) and combinations of the keywords according to the following search strategy: "Corona or coronavirus or COVID or COVID-19 or viral or virus " AND "nutrient or flavonoid or Quercetin”.

## Results

We proposed and hypothesized that quercetin, as a natural and harmless anti-viral, anti-inflammatory, antioxidant, and immunomodulatory flavonoid agent, can be used in combination with other compounds for reduction of the risk of developing some symptoms of COVID-19. This statement is based on quercetin inhibitory effect on SARS-CoV-2 main protease and RNA-dependent RNA polymerase.

Flavonoids, a large group of plant pigments, possess multiple subclasses such as flavones, flavonols, isoflavones, and chalcones (Panche et al., 2016). Some studies on the properties of flavonoids exhibited their anti-inflammatory, antioxidant, immunomodulatory, anti-allergic, anti-cancer, antifungal, antibacterial, and antiviral activities (Kumar and Pandey, 2013). Flavonoids including naringenin, naringin, kaempferol, apigenin, catechin, hesperetin, and quercetin indicated a wide range of antiviral activity against various viruses (Kaul et al., 1985). *In silico* studies suggested that flavonoid-based molecules may attenuate ACE-2 receptor activity by binding to its spike protein, helicase, and protease site (Guerrero et al., 2012). Overall, flavonoids like quercetin may be considered effective agents against SARS-CoV-2.

**Quercetin**

Quercetin, chemically named 3,3',4',5,7‑pentahydroxyflavone, belongs to a subgroup of flavonoids called flavonols (David et al., 2016). Quercetin, known as the sugarless form of rutin, is one of the most abundant flavonols in herbal foods (Lakhanpal and Rai, 2007). Quercetin is a yellow and crystalline solid with a bitter taste, which is widely used to reduce the risk of developing some symptoms of metabolic and inflammatory disorders. Oral bioavailability of quercetin is low with estimations that only 20% of the administered dose reaches the blood (Li et al., 2016). Maximum plasma concentration (C_max_) of quercetin was 2.3±1.5 μg/ml after oral administration at a dosage of 200 mg. Oral administration of quercetin for 12 weeks resulted in the highest concentration of quercetin in the lungs of the treated rats compared with the control group (Batiha et al., 2020). Quercetin undergoes phase II metabolism after absorption by the intestine (Almeida et al., 2018) and is metabolized to glucuronidated, sulfated, and methylated compounds in enterocytes or hepatocytes. Within 48 hours after absorption, quercetin is excreted into bile and urine as glucuronide or sulfate conjugates (Williamson et al., 2018). 

As a supplement, it appears to be generally safe with little to no side effects. Pharmacokinetic studies have demonstrated no adverse effects after up to 1 g/day oral supplementation with quercetin in human subjects (Colunga Biancatelli et al., 2020). In some instances, taking more than 1,000 mg of quercetin per day may cause mild symptoms like headaches, stomach aches, or tingling sensations (Ferry et al., 1996). Numerous studies have disclosed anti-inflammatory, antioxidant, immunomodulatory, antiobesity, antihypertensive, and antihypercholesterolemia, as well as antiviral activities of quercetin (David et al., 2016). 

**Antiviral effects of quercetin**

Various flavonoids are supposed to employ some activities against symptoms of different types of viruses through their interaction with the immune system (Lalani and Poh, 2020). As a flavonol from the flavonoid group of polyphenols, the antiviral effect of quercetin was investigated *in vitro* and *in* *vivo* studies against some viruses including hepatitis C virus (HCV), hepatitis B virus (HBV), influenza A virus (IAV), Mayaro virus (MAYV), dengue virus (DENV), human immunodeficiency virus (HIV), Japanese encephalitis virus (JEV), and porcine epidemic diarrhea virus (PEDV) (Di Petrillo et al., 2022). Also, quercetin administration (0 to 100 µM) resulted in inhibiting the replication and entry of Ebola virus (Fanunza et al., 2020). 

In addition, quercetin glucoside (200 µM) exhibited *in vitro* antiviral activity against multiple wild types of the Ebola virus. In mice supplemented with quercetin glucoside (50, 100, and 200 mg/kg of body weight), the Ebola viruses were blocked at the entry or post-entry step (Qiu et al., 2016). Lopes et al. (Lopes et al., 2020) demonstrated that quercetin pentaacetate incubation (10 µM) inhibited human respiratory syncytial virus (HRSV) adhesion on the surface of human epithelial type 2 (HEp-2) cells. Furthermore, treatment of HBV-infected HepG2 cells with quercetin (6.25, 12.5, 25, and 50 µg/ml) resulted in down-regulation of virus replication (Parvez et al., 2020) . 

An *in vitro* study evaluated the effect of quercetin (1, 10, 25, 50, 100, and 200 μmol/L) on HBV-infected cells and demonstrated a reduction of Hepatitis Bs antigen (HBsAg), Hepatitis B e antigen (HBeAg), and HBV genomic DNA levels (Cheng et al., 2015) . Administration of 100 µg/ml quercetin glucoside to influenza virus-infected Madin-Darby canine kidney (MDCK) cells prevented virus replication and had a suppressive effect on generating virus-induced cellular reactive oxygen species (ROS) (Nile et al., 2020) . Likewise, quercetin incubation (200 µM) in IAV-infected MDCK cells inhibited virus replication and regulated the expression level of genes of heat shock proteins and ﬁbronectin 1 (Vaidya et al., 2016) . Furthermore, viral RNA polymerase was inhibited by quercetin glucoside (10 µg/ml) in influenza A and B virus-infected MDCK cells (Gansukh et al., 2016). 

In IAV-infected MDCK cells incubated with quercetin (0 to 200 µg/ml), hemagglutinin (HA) mRNA transcription and nucleoprotein (NP) protein synthesis were reduced in a dose-dependent manner (Wu et al., 2015) . Orally administered quercetin (1 mg) reduced elevated levels of lipid peroxidation in viral infections and also protected against virus-induced oxidative stress in IAV-infected mice (Ruansit and Charerntantanakul, 2020) . Moreover, oral supplementation with 1 mg/day quercetin for 5 days contributed to the protection of the lungs against the deleterious effects of oxygen-derived free radicals released during influenza virus infection (Kumar et al., 2005) . Quercetin (6.25 mg/kg) reduced the post-infection pulmonary oedemas, influenza-associated undesired outcomes and the risk of mortality in influenza virus-infected mice (Choi et al., 2012) . 

Dos Santos et al. (dos Santos et al., 2014)  investigated the effect of quercetin glycoside incubation (0 to 100 μg/ml) on MAYV-infected Vero cells and indicated that quercetin glycoside inhibited MAYV replication in a dose-dependent manner (dos Santos et al., 2014) . In addition, quercetin (100 µg/ml) decreased the copy number of JEV RNA in infected Vero cells (Johari et al., 2012) . An *in vivo* study examined the effect of quercetin supplementation (5, 10, 15, 20, 25 or 30 mg/kg of body weight) on mengo-virus-infected mice and reported antiviral response against mengo virus (Veckenstedt et al., 1987) . Additionally, the administration of quercetin rhamnoside to PEDV-infected Vero cells inhibited virus replication (Song et al., 2011) . In HCV-infected Huh-7.5 cells supplemented with 50 µM quercetin, viral genome replication and infectious HCV particle production were reduced (Rojas et al., 2016). Furthermore, quercetin (10 µg/ml) inhibited heat shock proteins which are essential for HCV replication (Bachmetov et al., 2012) . Besides, dissection of the viral life cycle and reduction of the viral protein accumulation were caused by quercetin (50 µM) in HCV-infected Huh-7.5 cells (Gonzalez et al., 2009) . A summary of the studies on the antiviral effects of quercetin is presented in Table 1.

**Table 1:** Summary of studies on the antiviral effects of quercetin

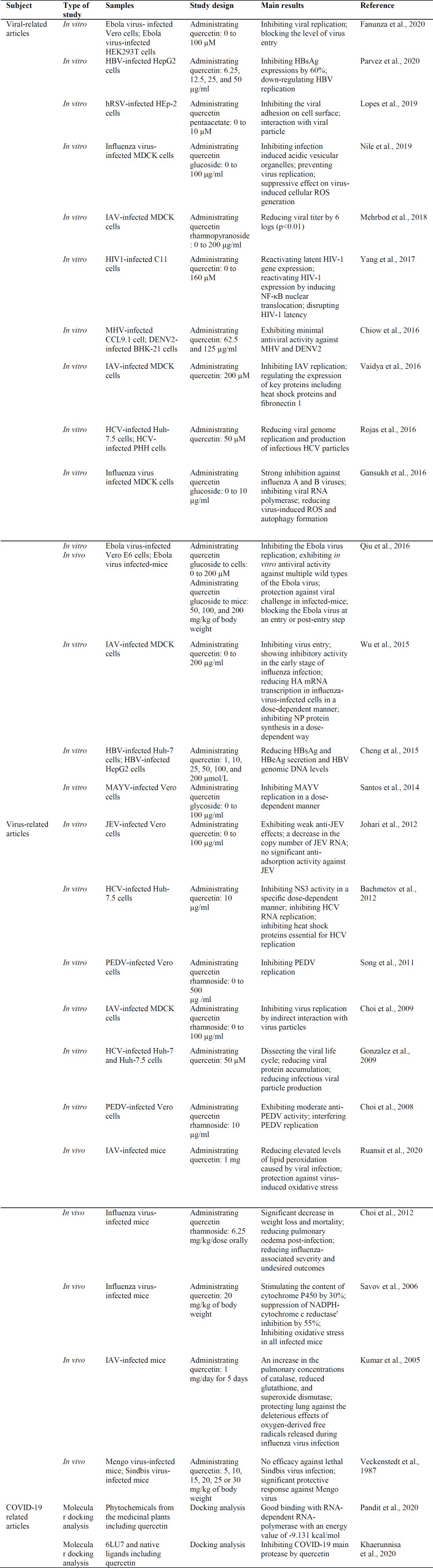

BHK, baby hamster kidney; DENV, dengue virus; HA, hemagglutinin; HBV, hepatitis B virus; HBeAg, hepatitis B e antigen; HBsAg, hepatitis B surface antigen; HCV, hepatitis C virus; HEp-2, human epithelial type 2; HIV, human immunodeficiency virus; hRSV, human respiratory syncytial virus; IAV, influenza A virus; JEV, Japanese encephalitis virus; MAYV, Mayaro virus; MDCK, Madin-Darby canine kidney; MHV, murine hepatitis virus; NF-κB, nuclear factor kappa B; NP, nucleoprotein; PEDV, porcine epidemic diarrhea virus; PHH, primary human hepatocytes; ROS, reactive oxygen species; Vero cells, African green monkey kidney cells.

**Anti-inflammatory and antioxidant activities of quercetin**

The second antioxidant line of body defense systems includes antioxidants such as quercetin (Tan et al., 2018) that scavenges free radicals, binds transition metal ions, and inhibits lipid peroxidation (Xu et al., 2019). Quercetin may prevent oxidation chain initiation and propagation (Mlcek et al., 2016). Moreover, quercetin may reduce irreversible damage to cell membranes by inhibiting inducible nitric oxide synthase activity (Shutenko et al., 1999). Xanthine oxidase pathway plays a key role in ischemia-reperfusion injuries caused by oxidative stress (González-Montero et al., 2018). Quercetin was reported to decrease oxidative injury through inhibiting xanthine oxidase activity (Chang et al., 1993) and can stabilize cell membrane action as a calmodulin antagonist (Baghel et al., 2012).

**Figure 1:**
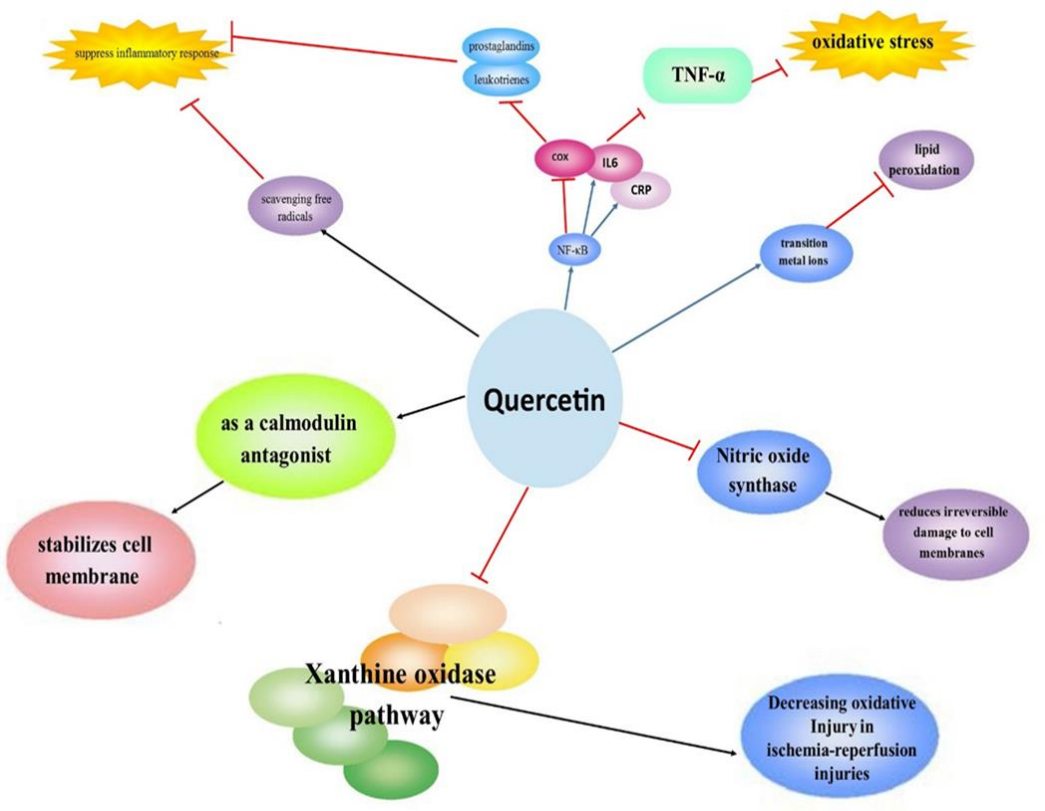
Anti-inflammatory and antioxidant activities of quercetin: Quercetin binds transition metal ions and inhibits lipid peroxidation. Quercetin reduces irreversible damage to cell membranes by inhibiting inducible nitric oxide synthase activity. Quercetin decreases oxidative injury through inhibiting xanthine oxidase. As a calmodulin antagonist it stabilizes cell membrane action acting. Quercetin can suppress inflammatory response by scavenging free radicals. NF-κB triggers the expression of several pro-inflammatory cytokines including IL-6, CRP, and COX-2 and reduces TNF-α generation. Inhibition of inflammatory enzymes such as COX by Quercetin decreases prostaglandins and leukotrienes.

Free radicals stimulate pro-inflammatory cytokines gene expression which is elevated in patients with chronic inflammatory diseases (Conner and Grisham, 1996). Quercetin can suppress inflammatory responses by scavenging free radicals (David et al., 2016). Tumor necrosis factor-alpha (TNF-α) is one of the main inflammatory biomarkers which is regulated by oxidative stress (Chen et al., 2008). Quercetin significantly reduced TNF-α generation and gene expression by attenuating the activation of the nuclear factor kappa B (NF-κB) pathway. NF-κB triggers the expression of several pro-inflammatory cytokines including interleukin-6 (IL-6), c‑reactive protein (CRP), and cyclooxygenase-2 (COX-2) (Granado-Serrano et al., 2012). Higher levels of COX-2 and NF-κB lead to the release of prostaglandins within the brain which regulate several central nervous system effects such as respiratory depression, fever, and pain (Aliabadi et al., 2020). Quercetin decreases inflammatory mediators including prostaglandins and leukotrienes through the inhibition of inflammatory enzymes such as COX and lipooxygenase (Yahfoufi et al., 2018). Also, the findings obtained from *in vitro*, *in vivo*, and human studies demonstrated that one of the most considerable properties of quercetin is its ability to modulate inflammation (Li et al., 2016). A recent randomized, controlled trial reported that supplementation with quercetin may lead to a higher decrease in CRP and ferritin levels and a higher increase in platelet and lymphocyte counts (Önal et al., 2021) (Figure 1).

**Immunomodulatory impact of quercetin**

Quercetin may potentially modulate immune system responses. Incubation of cultured blood peripheral mononuclear cells with quercetin contributed to the stimulation of T-helper cells to release Th-1-derived interferon-γ (IFN- γ). Also, Th2-derived IL-4 was reported to be down-regulated by quercetin incubation (Nair et al., 2002). In a murine model of asthma, quercetin modulated leukocyte biology by affecting Th1/Th2 balance (Park et al., 2009). Quercetin may attenuate antigen-specific T cell activation by reducing lipopolysaccharide (LPS)-stimulated dendritic cell activity (Huang et al., 2010). Studies that investigated the impacts of quercetin on immune system function exhibited inhibition of cytotoxic lymphocyte function and IL-6 production in LPS-stimulated neutrophils (Liu et al., 2005). Also, the expression level of Th2 cytokines such as IL-4 and IL-5 were regulated by quercetin (Jafarinia et al., 2020).

Moreover, *In vivo* immunonutrition studies demonstrated an improvement of natural killer (NK) cell lytic activity, neutrophil chemotaxis, and lymphocyte proliferation by quercetin administration (Álvarez et al., 2006){Álvarez, 2006 #2479;Burkard, 2017 #2480}. 

**Possible mechanisms of action of quercetin against COVID-19**

Researchers have recommended quercetin as an effective agent in managing SARS because of its antiviral activity against several members of the Coronaviridae family (Colunga Biancatelli et al., 2020). Quercetin administration (83.4 µM) resulted in an inhibition of SARS-coronavirus entry into Vero E6 cells (Yi et al., 2004). SARS-CoV-2 main protease or 3-chymotrypsin*-*like protease (3CLpro) plays a key role in viral replication due to its proteolytic activity (ul Qamar et al., 2020). Quercetin and its derivatives can bind to glutamine (Gln189) as an important site on 3CLpro and, thereby, blocking its activity (Chen et al., 2006). The binding energy obtained from docking the main protease of coronavirus with quercetin was -8.47 kcal/mol. Therefore, quercetin is one of the most suggested compounds due to its strong inhibition of SARS-CoV-2 main protease, which should be assessed in further studies (Khaerunnisa et al., 2020). 

As mentioned above, attenuation of the activity of ACE-2 receptors may help to manage COVID-19 (Liu et al., 2020a). Interaction between ACE-2 and the viral spike protein results in infection (Mathewson et al., 2008). Recently, a crystal structure between the viral spike protein and ACE-2 is discovered (Lan et al., 2020). *In silico* modeling recognized quercetin as a suitable option in disrupting SARS-CoV-2 viral spike protein and ACE-2 interactions (Williamson and Kerimi, 2020). Current evidence has demonstrated that natural compounds with RNA-dependent RNA polymerase inhibitory activity can be considered effective agents against COVID-19 complications (Zhu et al., 2020). RNA-dependent RNA polymerase or RNA replicase is a viral enzyme that is crucial for the viral RNA replication in host cells (Venkataraman et al., 2018). Quercetin can potentially bind to RNA-dependent RNA-polymerase with an energy value of -9.131 kcal/mol which leads to attenuating its activity (Pandit and Latha, 2020) . 

## Discussion

Experimental and clinical approaches can be used to test the hypothesis of the inhibitory effects of quercetin against COVID-19. The beneficial effects of quercetin should be independently investigated and confirmed in future longitudinal human studies.

 To examine the hypothesis, it is critically important to prove the quercetin inhibitory activity on SARS-CoV-2 protease and reducing ACE-2 receptors' activity through bioinformatics projects. Moreover, more specific identification and confirmation of the quercetin role in SARS-CoV-2 inhibition must be performed as discussed in previous studies. Although the data discussed in this study support the proposed hypothesis, future population-based studies are recommended to investigate the role of quercetin in reduction of developing some symptoms of COVID-19. 
